# ‘Simply my own’

**DOI:** 10.4102/ajod.v3i1.116

**Published:** 2014-04-01

**Authors:** Kobus Moolman

**Affiliations:** 1 Department of English Studies, University of KwaZulu-Natal, South Africa

‘The disabled self is always a reader of his or her own body.’ (Amber DiPietra)

Poetry about disability (written by men and women with disabilities) has suffered from a multiplicity of forms of misrepresentation and ignorance. The popular perception of disability poetry (indeed the popular perception of poetry in general) has favoured the sentimental and the moralistic. This is what I call Hallmark poetry; poetry that is determined to impart some type of inspirational lesson to the reader. And what better lessons than those that men and women with disabilities (preferably silently!) impart: courage, stoicism, patience in the face of tragic misfortune.

The American anthology *Beauty is a verb: The new poetry of disability* (2011) explodes this stereotype of poetry, of poetry about disability, and of disability. This ground-breaking literary collection testifies to a uniquely vital strain of poetry, one that despite its distinctive historical characteristics (in this book at least: modern America) nevertheless helps re-define the global field of disability studies and contemporary literary theory.

Their circumscribed readership notwithstanding, literary anthologies are able to bring together and describe the particular imaginative energies at work within a given moment, and thus transform the existing cultural debates and even spearhead a range of alternatives to the mainstream (both politically and aesthetically). I am thinking here of critical anthologies like Donald M. Allen’s *The new American poetry: 1945–1960* (1960), Jerome Rothenberg’s *Technicians of the sacred* (1968), and, closer to home, collections like *Voices from within: Black poetry from Southern Africa* (1982) by Michael Chapman and Achmat Dangor, and the more recent *It all begins: Poems from postliberation South Africa* (2002) edited by Robert Berold. What all such collections have in common is their ability to straddle disparateness (without flattening out or homogenising the field) and to enlarge rather than de-limit perceptions and experiences. *Beauty is a verb* is precisely one such collection.

In her preface to the book, Jennifer Bartlett argues for ‘explor[*ing*] not only what it means to have a genre called “Disability Poetics”, but to look at poetry influenced by an alternate body and how this intersection forms a third language’ (p. 15). In this very brief statement Bartlett brings together succinctly what I consider to be three key concerns not only of the whole collection itself, but of the contemporary discourse of disability studies too: the idea of alterity (or the non-normative), the idea of the body (or more explicitly, embodiment) and the notion of some synthesis (I use this term guardedly, aware of its deeper meaning) that points toward a new direction, what Michael Northen calls ‘a redefinition of beauty and of the way that disability is perceived’ (p. 21). At the heart of this collection, then — whether the poets aligned themselves openly with the aims of disability poetry or not — lies a challenge to the normalising discourses of embodiment, an interrogation of the socially constructed nature of our concepts of wellness, ability, beauty, even love. As poet Kenny Fries writes in his long poem ‘Beauty and variations’:

‘How else can I quench this thirst? My lips
travel down your spine, drink the smoothness

of your skin. I am searching for the core:
What is beautiful? Who decides?’ (p. 21)

*Beauty is a verb* is thus more than just a straightforward anthology of poetry. It is many things at once. Like Allen’s *The new American poetry: 1945–1960* it does seek to promote a new type of poetry, one that is slowly beginning to be recognised as a distinct genre in its own right (i.e. ‘Disability Poetics’) but like Allen’s collection, too, it includes important essays and statements by the poets themselves that help to map the field in a sometimes idiosyncratic, sometimes theoretical manner, but always deepening and challenging the reader’s sense of what ‘Disability Poetics’ might be, and so assisting the reader to understand the differences and the similarities between the various poets included in the collection.

Michael Northen, well known as the founder and editor of the disability poetics journal, *Wordgathering*, sketches in broad strokes a history of American Disability Poetry, outlining some of the key (if neglected) poets, activists and theorists who contributed to the movement, whilst acknowledging that:

Yet identifying oneself as a writer of disability poetry or even admitting the legitimacy of a body of work that could be called disability poetry itself is still a bridge that many poets themselves are reluctant to cross. (p. 23)

The book goes on then, in a chapter called ‘Early voices’, to feature and discuss the work of some of the early (mid-to-late 20th century and all sadly deceased) pioneers of the field: poets like Larry Eigner, Tom Andrews, Vassar Miller, Robert Fagan and Josephine Miles. Eigner, a cerebral palsied poet, does not address his disability directly, but in his form and layout on the page registers his attentiveness to the minutiae of everyday life seen from a stationary viewpoint. Andrews crosses genres rapidly, from memoir to letter to short lyric, all in his ‘Codeine diary’, whilst Fagan’s poem ‘Stiege’ is an extended meditation on motion (walking, climbing stairs) that shuttles back and forth between poetry and prose in a highly original manner.

The next section in the book, entitled ‘The disability poetics movement’, anthologises the work of poets who, as Bartlett describes it, ‘speak to a celebratory narrative of the non-normative body’. Jim Ferris — one of the chief theorists of what he calls ‘crip poetry’ — sings the body broken in true Whitmanesque fashion. Kenny Fries’ poetry is very interesting, although quite formal (making use of long two-line stanzas), the intensity of his writing combines plain speech with evocative, even sensuous renderings of the body that are both excitingly literal and metaphoric at the same time. Jillian Weise, like Fries, is another very strong poet. ‘The amputee’s guide to sex’ is darkly humorous, whilst ‘The old questions’ (‘*Do you sleep with it on?*’, ‘*Do you bathe with it on?*’, ‘*Will you take it off in front of me?*’) is confrontational, tender and poignant all at the same time.

The final two sections of the book, ‘Lyricism of the body’ and ‘Towards a new language of embodiment’, are almost indistinguishable from each other, at least for me. (This is not a criticism.) The first section ranges from very effective dryly humorous poems by Hal Sirowitz to formally innovative pieces by Laurie Clements Lambeth that echo the convulsive, staccato experience of multiple sclerosis. In her essay on disability and confessionalism, Sheila Black moves our stereotypical conception of this genre away from an almost prurient obsessiveness towards what I feel is the much more interesting, though more ambiguous, borderline between truth and fictionality:

As a poet, as a storyteller, I am attracted to the unruly and confrontational elements of the confessional, to the ways it complicates personal truth through a presentation that makes the audience continually question whether the speaker is to be trusted. (p. 205)

In her subtle and understated poetry Black successfully stages the tensions inherent in this liminal space, whilst the work of Stephen Kuusisto, who is blind, is also interested in notions of truth, but more after Emily Dickinson’s: ‘Tell the truth, but tell it slant.’

The final section in the book foregrounds the work of poets with disabilities whose work is concerned overtly with formal experimentation; as Bartlett writes in her preface, ‘rather than explaining an individual story, bodily condition is manifested through form’ (p. 17). There is insufficient space in this review to do justice to the strength of writing in this section (poets like C.S. Giscombe, Amber DiPietra, Bartlett herself and Danielle Pafunda); however, I must emphasise that the type of representation involved in this closing part goes far beyond a mere synergy between form and content. It points to a set of deeper relationships that traverse the inner and the outer, the real and the imaginary, language and the body, experience and thought. This is one of the radical breakthroughs that Disability Poetics has achieved, and one that demands recognition and study, particularly on the African continent. Disability Poetics has been able to take its natural and normal (!) involvement with embodiment and interfuse this with a range of contemporary literary styles from New Formalism to L-A-N-G-U-A-G-E Poetics, from the modern Long Poem to Life Writing, in order to arrive at a new connection, a new place. As Bartlett writes in ‘5 poems from autobiography’:

composed primarily
of water and light

this is my body
I am its light

a mere shadow remains
so that, the body is erased

excepting movement

I am all motion and
this motion is neither weak nor hideous

this motion is simply my own. (p. 305)

